# Fungi That Infect Insects: Altering Host Behavior and Beyond

**DOI:** 10.1371/journal.ppat.1005037

**Published:** 2015-08-06

**Authors:** Yanfang Shang, Peng Feng, Chengshu Wang

**Affiliations:** Key Laboratory of Insect Developmental and Evolutionary Biology, Institute of Plant Physiology and Ecology, Shanghai Institutes for Biological Sciences, Chinese Academy of Sciences, Shanghai, China; Geisel School of Medicine at Dartmouth, UNITED STATES

## Introduction

Approximately 1,000 species of the fungal phyla Microsporidia, Chytridiomycota, Entomophthoromycota (order: Entomophthorales), Basidiomycota, and Ascomycota are known to infect and kill insects [1]. Of these, species such as *Beauveria bassiana* and *Metarhizium robertsii* (Ascomycota: Hypocreales) are well-studied models for exploring the mechanisms of fungus–insect physiological interactions, and they are used as biological controls for insect pests [2]. Entomopathogenic Hypocreales are phylogenetically closely related to plant pathogens and endophytes [3], and their sexual stages belong to *Cordyceps* sensu lato [4]. However, these species are taxonomically diverse and differ from each other considerably in their genomic features [5–8] and, consequently, their host ranges, infection cycles, and life strategies. Fungal infection normally, but not always, results in the death of an insect in situ. The parasitic fungi such as the host-specific pathogen *Ophiocordyceps unilateralis* sensu lato can control insect brains and manipulate their behavior to reach death locations that are optimal for spore dispersal, the so-called fungal extended phenotype [9]. On the other hand, on top of physiological immune surveillance, insects (especially social insects such as ants and termites) can smell and avoid fungal pathogens, groom each other to clear pathogenic spores, generate a fever response, or die well away from their nestmates, which is termed behavioral or social immunity [10,11]. Either side of these interactions will benefit from behavioral changes in insects that maximize their adaptive fitness. In this paper, both types of insect behavior alternations are reviewed, and the underlying mechanisms are discussed.

## The Fungal Infection Cycle and Host Specificity

**Fig 1:**
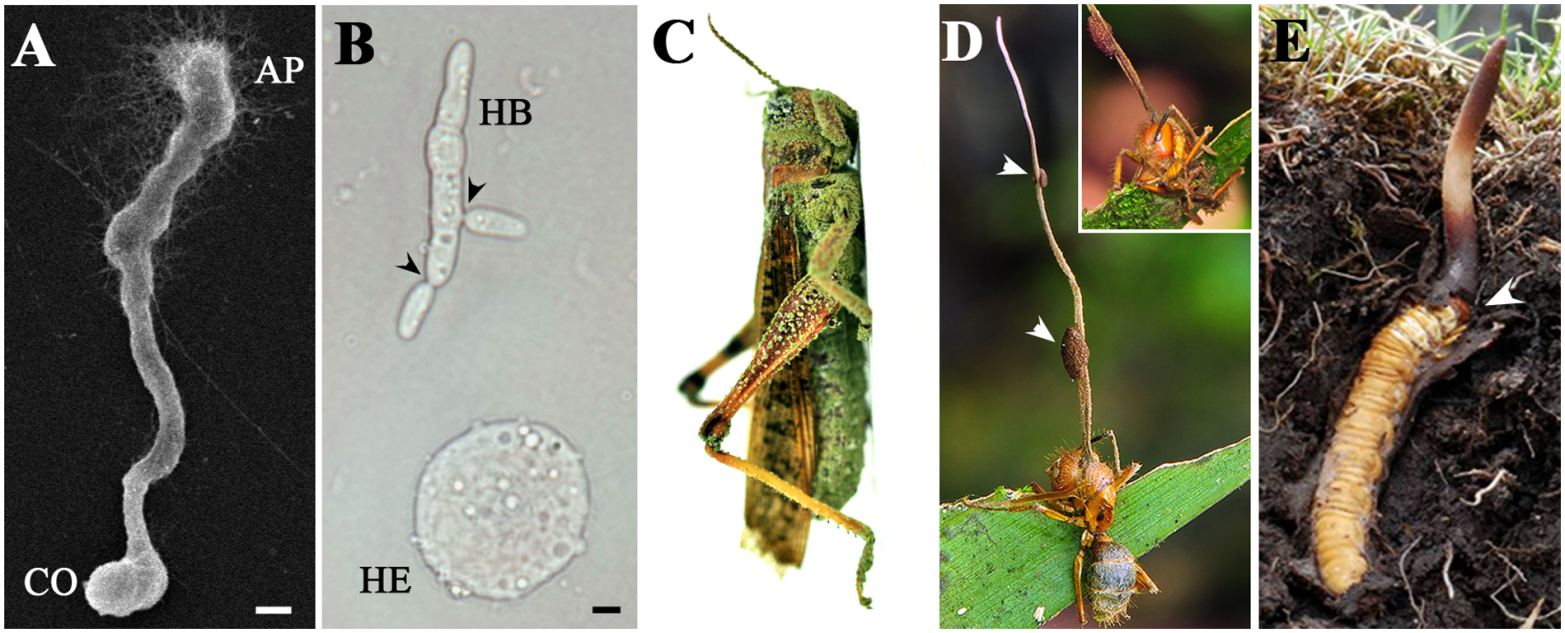
Micro- and macrophenotypes related to fungal infection and colonization of insect hosts. (A) Appressorium (AP) formation concurrent with mucilage production by *M*. *robertsii* on an insect cuticle 18 h after inoculation; CO: conidium; bar: 5 μm. (B) Formation of an *M*. *robertsii* hyphal body (HB) in a locust hemocoel 36 h after infection. Arrows point to the contraction rings formed for yeast-like budding; HE: hemocyte; bar: 5 μm. (C) Locust cadaver killed and mycosed by the asexual spores of the specific pathogen *M*. *acridum*. (D) Zombie ant. This carpenter ant (*Camponotus* sp.) was killed and colonized by *O*. *unilateralis* to form sexual fruiting body (stroma), which erupted from the insect’s head. The insert shows the front of the ant, which was holding tightly to the leaf before its death (courtesy of and copyright by Daniel Winker). Arrows indicate perithecial plates. (E) Caterpillar fungus. A ghost moth (*Hepialus* sp.) larva infected by *O*. *sinensis* remained close to the soil surface with its head up in death, and the stroma erupted from the insect’s head (arrow) (courtesy of and copyright by Daniel Winker).

Entomopathogenic fungi recognize and infect insects through the spore adhesion and formation of appressoria that penetrate the cuticle (Fig 1A). After reaching the hemocoel (body cavity) of an insect, fungal filaments will switch into yeast-like cells that undergo budding for rapid propagation and counteract the immune response of the hosts (Fig 1B). For the infection cycle to complete, dead insects must be either mycosed to produce asexual conidial spores (Fig 1C) or colonized to form a fruiting body (Fig 1D and 1E) to yield sexual spores for the next infection. Alternatively, insect pathogens such as *M*. *robertsii* with a broad host range can form a root–rhizosphere relationship to transfer nitrogen from dead insects to a plant and acquire carbon in return [12]; this is a strategy for long-term persistence in the soil when potential hosts are absent.

Different species of parasitic fungi have different insect host ranges. Generalist species, such as *B*. *bassiana* and *M*. *robertsii*, can infect hundreds of insect species of different orders, whereas species such as *M*. *acridum* (specific to locusts and grasshoppers) [3,5], *Cordyceps militaris* (specific to caterpillars) [6], and *O*. *unilateralis* sensu lato (specific to formicine ants), only infect a narrow range of insects [13,14]. In particular, *O*. *unilateralis* is a species complex that includes different species being highly host specific, which is close to a level of one fungus versus one ant species [13–15]. In terms of fungal host-specificity evolution, the study of *Metarhizium* species with different host ranges has shown a directional trajectory of speciation from being specialists to becoming generalists, and the process has been coupled with protein family expansions [3]. In particular, the number of divergent G-protein coupled receptors is significantly correlated with host specificity [3,5].

## Control of Insect Behavior by Parasitic Fungi

Parasites often manipulate the behavior of their hosts; for example, crickets or grasshoppers commit suicide by drowning when infected by the parasitic hairworm *Spinochordodes tellinii* [16]. A striking death grip behavior has also been observed in carpenter ants (*Camponotus* spp.) following infection by the parasitic fungus *O*. *unilateralis* [9]. The infected, moribund ants essentially behave like zombies; they walk alone and erratically climb to a certain height in the vegetation (approximately 25 cm above the soil surface). They bite leaf margins in rainforests and twigs in temperate woods and transition from wandering to biting takes place synchronously around noontime (within 11:00–14:00 h) possibly in association with a solar cue [13,17]. The fruiting body then erupts from their heads (Fig 1D). Likewise, the caterpillar fungus *O*. *sinensis* specifically infects the larvae of soil-dwelling ghost moths (*Hepialus* spp.) and maintains a symbiotic relationship for up to five years [8]. Before mummifying its host, the fungus drives a larva close to the surface of the soil (approximately 1–3 cm), and the fruiting body then grows from the caterpillar’s head (Fig 1E). Infections by obligate Entomophthorales pathogens could also lead to similar “summit” diseases, i.e., the infected insects climbing to an elevated position before death [18]. Such pathogen-controlled behavioral changes benefit the fungi by maximizing spore transmission efficiency to begin the next infection cycle.

## Inducement of Insect Behavioral Immunity

In contrast to being passively manipulated, insects can actively avoid and combat parasitic fungal infections through a behavioral or social immune response [19]. This especially occurs in social insects such as honeybees, ants, and termites, which can more easily compromise their relatively fewer antimicrobial peptide genes than nonsocial insects [20,21]. The hygienic behaviors of honeybees, ants, and termites, such as mutual grooming, can be triggered by the odor of *Metarhizium* fungal spores and provide behavioral resistance to the infection [22,23]. Other studies have indicated that ant allogrooming is coupled with chemical disinfection through the emission of formic acid, which reduces the viability of *Metarhizium* spores [24]. In termites, a salivary pleiotropic protein containing both a pattern recognition receptor and a β(1,3)-glucanase antimicrobial effector domain is used as a nest-building material to protect colonies against *Metarhizium* or bacterial infection [25]. Behavioral prophylaxis can also occur through social withdrawal and death in isolation. For example, garden ants (*Lasius neglectus*) infected by *M*. *anisopliae* stay away from the brood chamber, and moribund ants cease social contact with their nestmates and leave their nests hours or days before death [23]. So, in contrast with the zombie ants manipulated by *O*. *unilateralis* (Fig 1D), dying away from the colony is an active and altruistic response of fungus-infected ants. Density-dependent prophylaxis also occurs in locusts, but desert locusts (*Schistocerca gregaria*) actively raise their body temperature to inhibit *M*. *acridum* infection [26]. In addition, elevated physiological immune-surveillance has been observed in gregarious locusts, which enabled the locusts to collectively become more resistant to *Metarhizium* infection than solitary individuals [27].

## Remaining Secrets behind the Fungal Manipulation of Insect Behavior

As indicated above, behavioral alterations during fungus–insect interactions are diverse, but generally, host-specific and obligate pathogens manipulate insect behavior while the spores of generalist species can trigger active host behavioral immunity. Particular efforts including comparative proteomics, transcriptomics, and metabolomics have been undertaken to tentatively unravel the molecular mechanisms underlying fungal hijacking diseases [13,28]. However, unlike the single gene of a baculovirus (*egt* [ecdysteroid UDP-glucosyltransferase]) that can inactivate the gypsy moth molting hormone and thereby promote the summit disease [29] and a protein tyrosine phosphatase (*ptp*) of baculovirus that functions as a virion structural protein to facilitate virus infection of insect brain tissues to induce enhanced locomotory activity (ELA) [30], the key factor(s) involved in the fungal control of insect behavior is still unknown. Our genome survey indicated that a viral *egt*-like gene is not present, but a single copy of PTP domain-containing protein-encoding gene exists in the genomes of insect-parasitizing fungi, e.g., MAA_02506 of *M*. *robertsii* and BBA_03722 of *B*. *bassiana*. It remains to be determined whether the interference with hormone turnover or virus-like PTP-mediated ELA effect occurs during fungal infections. In addition, the genomes of parasitic fungi encode an array of lineage-specific, effector-like polypeptides as well as the gene clusters involved in the biosynthesis of small metabolites [3]. It has been found that the insecticidal cyclopeptide destruxins produced by *Metarhizium* could be used by the fungus to evade insect immunity, and its ability to produce a toxin is connected with host specificity [31,32]. The findings suggest that fungi could deploy small chemicals to alter insect physiology.

As in mammals, the dopamine (DA; a catecholamine neurotransmitter)-signaling pathway plays a conserved role in the modulation of invertebrate behavior [33]. Coupled with cuticle melanization, the up-regulation of DA-biosynthetic genes in the head of a locust coincides with the behavior change from solitary to gregarious [34]. DA has also been shown to contribute to behavior change and social interactions in ants [35]. An injection of biogenic amines into red wood ants (*Formica polyctena*) demonstrated that serotonin could stimulate aggressive behavior, while DA administration triggered mandible-opening and biting behaviors directed at foreign insects [36]. As indicated above, the mandibles of zombie ants usually penetrate deeply into plant tissues [9], so it is likely, although unconfirmed, that fungal infection could alter the accumulation of DA and other chemicals in insects, thereby causing precise behavioral manipulation.

## Future Directions

Alterations of host behavior during fungus–insect interactions are diverse, intricate, and of great scientific interest. Passive or active behavioral changes in insects are reminiscent of evolutionary adaptations that either promote cross-kingdom control by fungi or altruistic behavior by the hosts. There are still significant gaps in our understanding of the molecular mechanisms underlying behavioral alterations in insects during their interactions with fungi. Due to the taxonomic diversity of both insects and fungi, the molecular machinery involved in insect behavior changes could vary among the interacting species pairs or function on a case-by-case basis. The establishment of an ideal model system (e.g., the zombie ant), the acquisition of genomic information, and the deployment of the knowledge and techniques of the sciences of fungal genetics, secondary metabolism, chemistry, and insect physiology and neurology would help uncover the biological secrets behind changes in insect behavior.
